# Nutrient Intake Adequacy from Food and Beverage Intake of US Children Aged 1–6 Years from NHANES 2001–2016

**DOI:** 10.3390/nu13030827

**Published:** 2021-03-03

**Authors:** Ariana D.L. Bailey, Victor L. Fulgoni III, Neil Shah, Ashley C. Patterson, Fabiola Gutierrez-Orozco, Rebecca S. Mathews, Kelly R. Walsh

**Affiliations:** 1 Medical and Scientific Affairs, RB|Mead Johnson Nutrition Institute, Evansville, IN 47721, USA; neil.shah@rb.com (N.S.); ashley.patterson@rb.com (A.C.P.); fabiola.gutierrez-orozco@rb.com (F.G.-O.); kelly.walsh2@rb.com (K.R.W.); 2 Nutrition Impact, LLC, Battle Creek, MI 49014, USA; vic3rd@aol.com; 3 Gastroenterology Department, Great Ormond Street Hospital, London WC1N 3JH, UK; 4 R Mathews & Associates, Hudson, OH 44236, USA; rebecca.s.mathews@gmail.com

**Keywords:** NHANES, young children, nutrient adequacy, nutrient status, iron

## Abstract

The early years, between the ages of one and six, are a period of rapid physical, social and cognitive growth and a nutritionally adequate diet is an important factor for optimum development. We investigated the micronutrient adequacy and status of young US children aged 1–6 years (*n* = 9848) using 24-h dietary recall interviews completed by parents and caregivers participating in the National Health and Nutrition Examination Survey (NHANES) 2001–2016. data. The proportion of the sample not meeting the Dietary Reference Intakes (DRI) increased with increasing age and was most pronounced for calcium. Despite adequate iron intake, 7.4% and 2.5% had signs of iron deficiency and anemia based on serum ferritin and hemoglobin levels, with younger children and WIC participants at most risk and Non-Hispanic Black children the least. Vitamin B6 intake was adequate, but 6.4% had serum pyridoxal-5-phosphate deficiency. For vitamin E, 69% had intakes below the estimated average requirement (EAR), yet serum deficiency was only detected in 0.9%. Vitamin D intake was inadequate for 87%, but true deficiency may be overestimated. Mean DHA intake was 24 mg/d, well below expert recommendations of 70–100 mg/day. Iron and vitamin B6 deficiency and inadequate calcium, fiber, choline, potassium and DHA intakes are a concern for a significant percentage of young children. The discrepancy between nutrient intakes and serum deficiency levels needs to be further investigated.

## 1. Introduction

Early childhood between 1 and 6 years of age is characterized by growth spurts and rapid cognitive and social development [1]. A balanced diet is a factor necessary to ensure this occurs optimally. The requirements for macronutrients and micronutrients are higher for children compared to adults on a per-kilogram basis [2]. Although required in small amounts, micronutrients are critical for healthy development, maintenance and function of cellular processes, and overall wellbeing [3]. Iron intake during the toddler years of 1–3 years is particularly important due to decreased iron stores compared to infancy [4]. Adequate iron intake is needed for children’s growth, brain development, and immune function [5,6]. The consumption of specific long-chain fatty acids, such as docosahexaenoic acid (DHA) is also important for brain development. Although primary brain growth occurs from the prenatal period to about 2 years of age [7,8], the brain, particularly in the frontal lobes, continues to grow throughout the preschool years [9,10] Milk is the main source of energy in the diets of children aged 1–2 years [11] who are gravitating towards the intake of more solid foods, whereas children aged 3–6 years are transitioning more towards the family diet [2]. Both these changes may make children in this age range vulnerable to micronutrient inadequacy and deficiency. During this period children may also develop selective food preferences that eliminate certain foods and or food groups [12]. Recent food group intake estimates indicate mixed dishes, and more specifically the subcategory of burgers and sandwiches (including tacos and burritos) is the highest contributor to energy intake among Americans aged 2 years and older [11]. The second largest contributor to energy intake among children aged 2 to 19 years is snacks and sweets [11]. The widespread consumption of high energy foods with a lower concentration of essential nutrients makes it important to assess if the nutrient intake of young children is nutritionally adequate. 

Family income or socioeconomic status may also impact the nutrient adequacy of diets. A large body of observational study data have correlated lower socioeconomic status with nutrient-poor or energy-dense diets [13]. Studies show that the intake of sugary drinks, sweet or salty foods with high energy density is higher among children and adolescents when the socioeconomic status of the parents is lower [14]. Similarly, consumption of fruit, vegetable and dairy is higher when the socioeconomic status of the parents is higher [14]. Differences in race or ethnicity may also contribute to inadequate nutrient intakes. An evaluation of children aged 1–4 years who were participating in the Special Supplemental Nutrition Program for Women, Infants, and Children (WIC) reported that compared to Non-Hispanic White children, Hispanic children had better nutrient intakes and lower energy dense diets, while Non-Hispanic Black children had poorer nutrient intakes [15].

To date only three U.S. studies have assessed the dietary micronutrient intake adequacy in a nationally representative sample of young children. One study reported on children aged 6 to 23 months [16] and two studies on children from birth to less than 48 months [17,18]. The micronutrient intake in toddler and preschool US children aged 1–6 years has not been previously assessed. Therefore, the objective of this study was to determine the micronutrient adequacy of diets consumed by children aged 1–6 years, using NHANES 2001–2016 data and to examine if there were differences based on age, race/ethnicity, and family income. A secondary objective was to determine nutrient status based on specific biomarkers. Micronutrient adequacy was assessed solely on the basis of food and beverage consumption, as the 2015–2020 Dietary Guidelines for Americans [19], as well as the American Medical Association [20] and other health professional groups recommend that vitamin and mineral needs be met primarily from foods and not from vitamin and mineral supplements.

## 2. Materials and Methods

### 2.1. Data Source, Usual Intake, Demographics. and Nutrient Biomarkers

NHANES is an ongoing annual cross-sectional survey conducted by the National Center for Health Statistics at the Centers for Disease Control and Prevention to assess the nutritional status and health of U.S. children and adults. It uses stratified multistage probability cluster sampling to ensure adequate representation of the U.S. population [21]. The survey consists of an interview of demographic, socioeconomic, dietary and health-specific questions, as well a physical examination that includes medical, dental, physiological, and laboratory measurements [21]. All relevant information, including dietary intake is provided by a parent or proxy for children under 5 years [16,22]. For children aged 6 years, a parent or proxy assists with the interview and dietary intake assessment [22]. 

Direct measurement of usual intake for long periods is difficult to obtain in large population surveys [23]. Therefore, NHANES developed analytical techniques to estimate usual intake using 2 days of 24-h dietary recalls [22]. The first dietary recall is administered in person and the second recall is administered by phone 3 to 10 days later [16,22]. The interview is conducted by a trained interviewer who records all foods and beverages consumed in the 24-h period before the interview with the assistance of a computerized interview system with standard probes to reduce the probability of missed or forgotten foods in a multiple-pass method, i.e., USDA’s Automated Multiple-Pass Method [16,22].

The NHANES 2001–2016 data set of nutrient intake from foods and beverages was used to estimate usual intake for children aged 1–6 years as a whole, as well as sub groups of 1–3, 4–6 years, with additional focus on children 1–2 and 2–3 years which is when DHA and iron intake is of greater importance. In the data set, socioeconomic status was defined by the poverty income ratio (PIR), an index of family income in relation to need as determined by a federal poverty guideline (16) as well as by eligibility and participation in WIC. PIR was categorized in three groups: <1.35, 1.35–1.85, and >1.85. The NHANES 2011–2016 data set was used to assess usual nutrient intake by ethnicity, as it provides the most recent self-reported ethnic classifications of Non-Hispanic White, Non-Hispanic Black, Hispanic, and Asian individuals. When ethnic sample numbers were too low in the NHANES 2011–2016 data set, the NHANES 2001–2016 data set was used with classifications of Non-Hispanic White, Non-Hispanic Black, Mexican American, and Other.

Nutrient status was measured using available biomarker data for the specified population. Those nutrient biomarkers included: iron (hemoglobin < 11 g/dL and ferritin < 12 ng/mL), folate (RBC folate < 95 ng/mL), vitamin A (retinol < 20 µg/dL), vitamin B6 (pyridoxal 5’phosphate < 20 nmol/L), vitamin B12 < 200 pg/mL, and vitamin E (α-tocopherol < 500 µg/dL).

### 2.2. Statistical Analysis

Nutrient values from foods and beverages consumed during each 24-h recall period were calculated from USDA’s National Nutrient Database for Standard Reference. The two 24-h recall days were used to assess usual nutrient intake using the National Cancer Institute (NCI) method [23], a mixed effects model and quantile estimation procedure that corrects for intra-individual variation across the two days. Estimated usual intakes were generated for vitamins A, C, D, E, B6, B12, thiamin, riboflavin, niacin, calcium, iron, phosphorus, magnesium, choline, sodium, potassium, zinc, fiber, and DHA. The usual intake of nutrients was compared to age specific Daily Reference Intakes (DRIs) established by the Institute of Medicine (IOM), namely the Estimated Average Requirement (EAR) and Adequate Intake (AI) using the cut point method to determine the percent of the population with inadequate intakes or above the AI. No EAR has been established for DHA, but two global expert bodies, the French Food Safety Agency (AFFSA) [24] and the European Food Safety Authority (EFSA) [25], recommend a range of 70–100 mg/d for children up to 3 years, which was used as a reference for comparing usual DHA intakes.

Stratified analyses by age (1–6, 1–2, 2–3, 1–3, 4–6), race/ethnicity (Non-Hispanic White, Non-Hispanic Black, Hispanic, and Asian), and PIR (<1.35, 1.35–1.85, >1.85) were performed. Significant differences among population proportions were determined using a Z-statistic. Logistic regression analyses were used to assess whether certain demographic groups were more or less likely to have nutrient biomarkers below recommended levels. Statistical significance in all analyses was accepted if *p* < 0.05.

## 3. Results

### 3.1. Micronutrient Adequacy

The data for 9848 children aged 1–6 years were analyzed from NHANES 2001–2016. Table 1 shows the demographics of the group. The proportion of girls and boys were equally distributed for all age groups. Thirty-eight percent of the sample lived in households that had an income less than 1.35% of the poverty level. Children in the 1–2 year-age group had a slightly higher percentage, (i.e., 40%) than other age groups to have a family income less than 1.35% of the poverty level. Non-Hispanic White children comprised half the sample, 25% were Hispanic children, about 15% were Non-Hispanic Black, and approximately 5% were Asian.

Table 2 shows the micronutrient intake of children aged 1–6 years and the proportion not meeting the DRIs. Almost all of the children aged 1–6 years met the EAR for most vitamins and minerals; however, 87%, 69%, and 17% had intakes below the EAR for vitamin D, vitamin E, and calcium, respectively. About 40% of the sample had intakes above the AI for choline and potassium, whereas almost 100% exceeded the AI for sodium. Less than 1% of the sample met the AI for fiber. Supplementary Table S1 provides a further breakdown by age group (i.e., 1–2 years, 2–3 years, 1–3 years, and 4–6 years). The proportion of children not meeting the EAR for vitamin D appeared to increase with increasing age: 79.2% for 1–2 years; 87.3% for 2–3 years; 90.8% for 4–6 years, with a significant difference between those aged 1–3 years versus 4–6 years (*p* < 0.05). There was a dramatic shift in calcium intakes below the EAR with increasing age, 3.6% for children aged 1–3 years and 30.4% among those aged 4–6 years (*p* < 0.05). Similarly, those that had potassium intakes above the AI decreased from 45% in children aged 1–3 years versus 30% among children aged 4–6 years (*p* < 0.05). The percentage of children with choline intakes above the AI also decreased from 52% to 27% when children aged 1–3 years were compared to children 4–6 years (*p* < 0.05). Correspondingly, fiber intakes above the AI decreased from approximately 1% in children 1–3 years to 0.2% in children 4–6 years (*p* < 0.05). 

There was evidence of differences in intake adequacy for some vitamins and minerals when ethnic groups among children 1–6 years from the 2011–2016 cycles were compared (Table 3). Vitamin D intakes below the EAR was most prevalent among Non-Hispanic Black children compared to the other groups (94.9% vs. 90.4% for Non-Hispanic White, 88.5% for Hispanic, and 80% for Asian (*p* < 0.05, all comparisons). Similarly, a greater number of Non-Hispanic Black children (26%) had calcium intakes below the EAR compared to Non-Hispanic White (14.3%), and Hispanic (12.5%) children (*p* < 0.05, all comparisons), whereas intakes were not significantly different from Asian children (16.5%). In contrast, Non-Hispanic Black children (40.1%) had the lowest percent of vitamin E intakes below the EAR compared to all three of the other groups (*p* < 0.05, all comparisons). Vitamin C inadequacy was higher among Asian children (6.8%) versus Non-Hispanic Black (0.4%), and Hispanic (0.1%) children (*p* < 0.05, all comparisons), but not significantly different from Non-Hispanic White children (2.7%). A higher proportion of Non-Hispanic Black children (35.8%) had potassium intakes above the AI compared to Non-Hispanic White children (26.4%) (*p* < 0.05), but not significantly different from Asian (33.4%) or Hispanic (37.6%) children. Asian (54.9%) and Hispanic (50.4%) children had greater percentage of choline intakes above the AI compared to Non-Hispanic White children (32.4%) (*p* < 0.05, all comparisons), but not significantly different from Non-Hispanic Black children (42%). Fiber intake was not significantly different among the four ethnic groups. In general trends were similar between ethnicities among different age groups (Supplemental Table S2a–d). The only noteworthy observations were that among 1–3 year children, vitamin E inadequacy was significantly higher in Hispanic children (60.6%) compared to Non-Hispanic Black children (43.8%) (*p* < 0.05) and among 4–6 years children, vitamin C inadequacy was significantly higher among Asian children (9.4%) versus Non-Hispanic Black (0.7%) and Hispanic (0.3%) children (*p* < 0.05, all comparisons). Among all age groups, a lower proportion of Non-Hispanic White children exceeded the AI for potassium and choline (*p* < 0.05, all comparisons). 

Classification by PIR did not appear to significantly impact vitamin and mineral intakes below EAR or those above the AI among children 1–6 years, when considered as a whole group (Table 4). One exception was choline intake; the percent of children above the AI in the PIR < 1.3 category (46.1%) was significantly higher than those in the PIR ≥ 1.85 category (34.6%) (*p* < 0.05). Analysis by age groups (Supplementary Table S3a–d) revealed that children aged 1–3 years who came from families with incomes below the poverty line (PIR < 1.3) had a higher prevalence of calcium inadequacy than those from families with incomes 1.85 times the poverty level (4.8% vs. 2.5%, *p* < 0.05). The proportion of children with choline intake above the AI was consistently higher by 11–12% in the lowest PIR category versus the highest (*p* < 0.05, all comparisons) in all age groups.

The usual mean intake of DHA was 24 mg/d for children aged 1–6 years with minimal difference in intake between younger and older children within this age range (Table 5). Compared to global recommendations of 70–100 mg/d, 97–99% of the sample had intakes below this level. Even the 90th percentile of DHA intake (44 mg/d) was significantly below the lower end of the recommended range.

### 3.2. Micronutrient Status

Serum biomarkers of iron, folate, and vitamins A, B6, B12 and E status were evaluated by using standard cut-off values for deficiency (Table 6). Vitamin C and D status were not evaluated, as NHANES 2001–2016 data did not include this information for children below 6 years of age; additionally, data for serum vitamin A and vitamin E were only available for children 2 years and older and 4 years and older, respectively. Less than 2% of children 1–6 years had biomarkers of folate, vitamins A, B12 and E below deficiency levels. A little over 6% had serum vitamin B6 levels below the deficiency cut-off. Iron status, as indicated by serum ferritin and hemoglobin, fared the worst of the biomarkers evaluated. For serum ferritin, which was used as the marker for iron deficiency, 7.4% of the children had serum ferritin levels below the cut-off of 12 ng/mL with 4.6% of children falling below the more conservative cut-off of 10 ng/mL. For hemoglobin, which was the indicator for anemia, 2.5% had levels below the deficiency level of 11 g/dL. Significant age differences were observed in the prevalence of children below the deficiency indicators for serum ferritin and hemoglobin. Among children aged 1–3 years, 10.7% had serum ferritin below 12 ng/dL compared to 3.7% among children aged 4–6 years (*p* < 0.05). Similarly, 3.6% of children aged 1–3 years had a hemoglobin concentration less than 11 g/dL compared to 1.6% among children aged 4–6 years (*p* < 0.05).

Logistic regression analysis was performed for biomarkers that had 2% or more of the population falling below deficiency levels (Supplemental Table S4). The NHANES 2011–2016 data indicated children aged 1–6 years who were Hispanic and Asian were 5.5 (*p* = 0.01) and 13.6 (*p* = 0.04) times more likely (*p* = 0.04) to have ferritin levels below 12 ng/mL than Non-Hispanic White children, whereas Non-Hispanic Black children had an equivalent risk. However, significant weight cannot be given to this analysis as only 20 children fell below the serum ferritin cutoff. NHANES 2001–2016 had 322 children aged 1–6 years with ferritin below 12 ng/mL and logistic regression against Non-Hispanic White children showed Non-Hispanic Black children had half the risk (*p* = 0.003), whereas no significant risk differences were observed when Mexican Americans, Hispanics, or Other groups were compared. These data also revealed that children who came from families that participated in WIC were 1.5 times (*p* = 0.03) more likely to have ferritin levels below the 12 ng/mL cut-off than those who were not WIC eligible. Male children also showed a trend towards having a 1.4 times increased probability of having ferritin levels below 12 ng/mL compared to female children (*p* = 0.06). The likelihood of having hemoglobin below 11 g/dL was more than three times higher in Non-Hispanic Black children versus Non-Hispanic White children (*p* < 0.0001); however, no effect of sex or WIC eligibility was observed. Ethnicity and sex also did not appear to impact deficiency levels of serum vitamin B6, however being WIC eligible increased the probability by two times compared to those who were not eligible (*p* = 0.03).

## 4. Discussion

Analysis of the NHANES 2001–2016 data revealed that US children aged 1–6 years have adequate nutrient intakes for the majority of micronutrients. Prevalence of inadequacy was low (≤1%) for vitamin A, thiamin, riboflavin, niacin, vitamin B6, folate, vitamin B12, vitamin C, iron, magnesium, phosphorus, and zinc. However, a significantly large proportion of this population sample had intakes below the EAR for vitamin D (87%), vitamin E (69%), and calcium (17%). Approximately 40% had intakes above the AI for choline and potassium, and less than 1% had intakes above the AI for fiber. The proportion of children not meeting the recommended intakes for vitamin D, calcium, fiber, choline, and potassium increased with increasing age. DHA intake was below the AFFSA and EFSA expert recommendations of 70–100 mg/day for nearly all children. Many of the shortfall nutrients in our analysis were similar to the findings of other researchers. Among toddlers aged 12–23 months from NHANES 2009–2012, 74% and 82% had vitamin D and vitamin E intakes below the EAR, respectively; only 49% had intakes above the AI for choline and less than 1% had intakes above the AI for fiber and potassium [16]. Intakes of vitamin D had the highest level of inadequacy among young children aged less than 48 months across FITS 2002, 2008, and 2016, followed by vitamin E [18]. Across age categories of 12–47.9 months, 76–80% and 30–52% of the children had intakes below the EAR for vitamin D and E, respectively when intakes from foods, beverages and dietary supplements were analyzed [17]. In the same study, 9% of children aged 12–23.9 months, 6.4% of children aged 24–35.9 months, and 9% of children aged 36–47.9 months had calcium intakes below the EAR [17]. The evidence here shows that a significant portion of this population do not meet the EAR for these key nutrients even when supplement use was taken into consideration. 

The most salient finding of our study was that despite adequate intakes of iron for a majority of the population, 7.4% of children aged 1–6 years had serum ferritin levels below 12 ng/mL, 4.6% had ferritin levels below 10 ng/mL and 2.5% had hemoglobin levels below 11 g/dL. Similar iron status findings were observed in a study that examined 2007–2010 NHANES data for children aged 1–5 years [26]. Iron deficiency was 7.1%, based on calculated total body iron values from transferrin receptor and ferritin concentrations [26]. Anemia was 3.9%, defined as a hemoglobin concentration less than 11 g/dL for children 6–59 months and <11.5 g/dL for children 60–72 months [26]. The prevalence of iron deficiency anemia was 1.1%, defined as having both anemia and iron deficiency [26]. We observed significant differences in children aged 1–3 years and 4–6 years for both parameters (ferritin: 10.7% vs. 3.7%; anemia; 3.6% vs. 1.6%, *p* < 0.05 for both). The 2007–2010 NHANES analysis found children aged 1–2 years versus children aged 3–5 years had higher prevalence rates of iron deficiency (13.5% vs. 3.7%, *p* < 0.05) and anemia (5.4% vs. 1.9%, *p* < 0.05) [26], similar to our results. No clear ethnic differences emerged in our data with the exception that Non-Hispanic Black children were less prone to deficiency ferritin levels. However, Non-Hispanic Black children were more than three times as likely to have anemia based on hemoglobin levels, suggesting that anemia present in these children were likely induced from causes unrelated to iron stores. Children who were enrolled in the WIC program were 1.5 times as likely to be iron deficient (defined by falling below the ferritin cut-off), compared to those that were not WIC eligible. This finding is consistent with low hemoglobin and hematocrit levels in children aged 1 to less than 5 years who participated in WIC from a national survey of WIC participants [27].

Based on the finding that only 1% of our study sample had iron intakes below the EAR, yet a significant proportion of the population were characterized as iron-deficient based on serum ferritin measures, suggests the possibility of some of the following scenarios: over-reporting of intakes, reduced bioavailability of the iron consumed, increased iron demand associated with rapid growth during early childhood, or an iron EAR that is too low for this age group. Dietary choices have an impact as non-heme iron found primarily in plant foods is markedly less bioavailable than heme iron found in animal food sources (5–10% vs. 20–30%) [28]. Non-heme iron absorption efficiency is linked to the iron status of the individual, the amount of bioavailable non-heme iron, and the balance between inhibiting (i.e., phytate in cereal products, legumes; polyphenols in cocoa, tea, some vegetables, herbs, nuts; calcium; soy protein; inositol-containing foods) and enhancing (i.e., ascorbic acid, meat, fish, seafood; certain organic acids such as citric, lactic, malic, tartaric) factors of iron absorption [28,29]. The type of iron used in iron-fortified products may also influence iron absorption rates [29].

Serum biomarkers for folate, vitamin B6, vitamin A, vitamin 12, and vitamin E data were available for this population. Less than 1–2% of the sample had serum deficiency levels of vitamin B12, folate and vitamin A, consistent with intakes measured by EAR that indicated these were not nutrients of concern. However, as in the case of iron, there were discrepancies between inadequacies measured by the EAR and actual serum levels for vitamin B6 and vitamin E. Vitamin B6 consumption was deemed adequate for most of the population, yet 6.4% had a serum pyridoxal-5-phosphate deficiency. There is evidence that in some ethnic groups, underweight individuals, and other population sub-groups, plasma pyridoxal-5-phosphate levels are low despite adequate or higher intake from supplements that are above the RDA [30,31]. These data suggest that the current DRI may be too low as it may not guarantee adequate vitamin B6 status in some population groups [30,31]. For vitamin E the converse was true, where 69% had intakes below the EAR, yet deficiency levels were only detected in 0.9% of the population, suggesting the possibility that the current EAR may be too high; however, data for this biomarker were limited to age 4 and greater.

Serum vitamin D NHANES 2001–2016 data was not available for this age group. As noted earlier, a high proportion of children aged 1–6 years have vitamin D intake from foods and beverages that are below the EAR. However, vitamin D from dietary supplements were not part of the assessment, and therefore this analysis may have overestimated the level of inadequacy. Furthermore, vitamin D is synthesized in the body when an individual is exposed to sunlight and varies based on racial differences and synthetic ability. Vitamin D status from blood samples of children aged 1–6 years participating in NHANES 2001–2004 showed true deficiency levels were much lower than would be anticipated, but that Non-Hispanic Black children (10%) were the most likely to be deficient (25-hydroxyvitamin D < 15 ng/mL) [32]. This is consistent with our finding that a significantly higher percentage of Non-Hispanic Black children aged 1–6 years have intakes lower than the EAR compared to the other ethnic groups. In the NHANES 2001–2004 analysis, deficiency data were not available for Mexican American children, but levels of insufficiency (15–29 ng/mL) were 43–48%, 60–79% and 75–76% for Non-Hispanic White, Mexican American, and Non-Hispanic Black girls and boys, respectively [32].

Currently there is no effective large-scale screening method to adequately assess calcium status in a population [33], and therefore one is limited to the estimation of intakes as a measure of calcium deficiency in this population. Calcium was identified as a shortfall nutrient in our analysis based on the percentage of children that fell below the EAR (i.e., 17%) and increasing age was associated with increased calcium inadequacy. It was a greater concern for children aged 4–6 years (30.4%) than for children aged 1–3 years (3.6%) (*p* <0.05). A greater proportion of Non-Hispanic Black (26%) had inadequate calcium intakes compared to Non-Hispanic White and Hispanic children (*p* < 0.05). Our results were based on food and beverage intake and therefore unclear to what extent calcium dietary supplementation impacted overall intake. As stated earlier, the FITS 2016 study found a significant portion of this population did not meet the calcium EAR even when supplement use was taken into consideration [18]; however, no clear age trend was observed in the FITS 2016 data versus our data that included older children and spanned 16 years. 

Almost the entire population (97–99%) of children aged 1–6 years had DHA intakes below the expert recommendations of 70–100 mg/d. The mean intake of 24 mg/d observed in our NHANES 2001–2016 analysis is consistent with the mean intake of 20.5 mg/d for children aged 1–5 years in an analysis of NHANES 2003–2008 [34]. Consistent with their finding was the low intake of fish, which are a high source of DHA, possibly due to concerns about mercury contamination and food allergies [34]. Data collected by the FAO has indicated that low DHA intake among infants and young children is a global phenomenon [35]. Although there has been increased recognition of the importance of DHA for normal brain function and particularly the increased need of DHA during infancy and spurts of development in early childhood [9], many policy makers have not yet formally developed recommended dietary intakes for DHA [35].

The strengths of our study include a large nationally representative sample of children 1–6 years that has used the standardized NCI usual intake methodology. To our knowledge, this is the first study that has examined the usual nutrient intakes and nutrient biomarkers of this age group. Limitations include the cross-sectional nature of the study, self-report of dietary intake of young children from parents/proxies, some of whom may not know the precise foods and quantities eaten when they were at work, small sample sizes of some sub-group analyses, and no assessment of the contribution of dietary supplement use to total nutrient intake.

## 5. Summary and Conclusions

In summary, this evaluation of NHANES 2001–2016 shows that the usual intakes of most micronutrients were adequate among toddlers and children aged 1–6 years, except for vitamin D, vitamin E, calcium, fiber, choline, and potassium. Although iron and vitamin B6 intakes appeared to be adequate for most of the population, a significant proportion (>5%) had iron and vitamin B6 deficiency based on serum ferritin and plasma pyridoxal-5-phosphate markers. A small percent also had anemia based on hemoglobin levels. In contrast, a large percentage of the population had vitamin E intakes below the EAR, yet serum deficiency was detected in less than 1% of the population. Vitamin D intake inadequacy, while highly prevalent, may have been overestimated as the impact of supplements and sunlight were not accounted for. Inadequate calcium intakes were higher in children aged 4–6 years than children aged 1–3 years and among Non-Hispanic Black children compared to Non-Hispanic White and Hispanic children. DHA intake was universally low in all of the children evaluated. Dietary intervention strategies can be implemented to address the nutritional gaps identified in this population. Additional research is needed in this age group to confirm and extend these findings.

## Figures and Tables

**Table 1:** Demographics of children 1–6 years by age group (NHANES 2001–2016) ^1^.

Characteristic	1–2 y	2–3 y	Total 1–3 y	4–6 y	Total 1–6 y
n ^2^	4162	3573	5579	4269	9848
Girls (%)	50.1 ± 1.1	50.3 ± 1.2	49.7 ± 0.9	49.2 ± 1.1	49.5 ± 0.7
Poverty Income Ratio (%) ^3^					
<1.3	40.4 ± 1.2	38.7 ± 1.4	39.4 ± 1.2	37.5 ± 1.4	38.4 ± 1.2
1.3–1.85	10.6 ± 0.7	11.4 ± 0.7	11.2 ± 0.6	10.7 ± 0.8	10.9 ± 0.5
≥1.85	49.0 ± 1.3	49.9 ± 1.6	49.5 ± 1.3	51.9 ± 1.5	50.6 ± 1.2
Race/Ethnicity (%) ^4^					
Non-Hispanic white	50.9 ± 3.6	51.1 ± 3.5	50. 9 ± 3.2	49.4 ± 4.0	50.1 ± 3.4
Non-Hispanic black	13.5 ± 1.9	14.8 ± 2.0	13.7 ± 1.8	15.7 ± 1.9	14.7 ± 1.7
Hispanic	26.0 ± 2.9	24.0 ± 2.8	25.4 ± 2.7	24.8 ± 2.9	25.1 ± 2.5
Asian	3.5 ± 0.7	4.1 ± 0.7	4.1 ± 0.7	4.8 ± 0.7	4.5 ± 0.5

^1^ Values are weighted percentages ± SEs unless otherwise indicated. Data are from NHANES 2001–2016, unless otherwise indicated. ^2^ Unweighted sample size (note both of the younger groups include those 2 years of age). ^3^ <1.3 refers to income less than 1.3 times the poverty level; 1.3–1.85 refers to income 1.3 to 1.85 times the poverty level; >1.85 refers to income greater than 1.85 times the poverty level. ^4^ Prior to 2011, ethnicities measured were non-Hispanic white, non-Hispanic black, Mexican American, Other Hispanic and Other; only race/ethnicities sampled to be representative are included thus total presented will not add to 100%. In 2011–2016, the designations are as specified in Table 1. Values for the category Other is not reported.

**Table 2:** Mean intakes of micronutrients for children 1–6 years and proportions not meeting Dietary Reference Intakes (NHANES 2001–2016).

Nutrient	Mean Intake ± S.E. ^1^	% Population < EAR ^2^	% Population > AI ^2^
Dietary Fiber (g)	11 ± 0.1		0.6 ± 0.1
Vitamin A, RAE (mcg)	563.9 ± 6.2	1.3 ± 0.3	
Thiamin (mg)	1.3 ± 0.0	0.0 ± 0.0	
Riboflavin (mg)	1.9 ± 0.0	0.0 ± 0.0	
Niacin (mg)	15.7 ± 0.1	0.1 ± 0.0	
Vitamin B6 (mg)	1.4 ± 0.0	0.0 ± 0.0	
Folate DFE (mcg)	428.5 ± 4.1	0.1 ± 0.0	
Vitamin B12 (mcg)	4.4 ± 0.1	0.0 ± 0.0	
Vitamin C (mg)	84.9 ± 1.5	1.3 ± 0.2	
Vitamin D (μg)	6.7 ± 0.1	86.6 ± 0.8	
Vitamin E (mg)	4.9 ± 0.1	69.2 ± 1.1	
Calcium (mg)	978.7 ± 8.9	16.8 ± 0.7	
Iron (mg)	11.6 ± 0.1	1.1 ± 0.1	
Magnesium (mg)	197.9 ± 1.2	0.4 ± 0.1	
Phosphorus (mg)	1092 ± 8	0.0 ± 0.0	
Choline (mg)	213.9 ± 2.1		39.7 ± 1.3
Sodium (mg)	2313 ± 15		99.8 ± 0.1
Potassium (mg)	2021 ± 14		37.8 ± 1.1
Zinc (mg)	8.5 ± 0.1	0.1 ± 0.0	

^1^ Values are means ± S.E. of nutrients from food and beverage intake during a single 24-h dietary recall completed by parents. ^2^ Determined via cut-point method using usual intakes as estimated using the National Cancer Institute method employing use of both dietary recalls compared to recommended intakes. AI: Adequate Intake; DFE: Dietary Folate Equivalent; EAR: Estimated Average Requirement; RAE: Retinol Activity Equivalent.

**Table 3:** Mean nutrient intake of children 1–6 years and proportion not meeting Dietary Reference Intakes by Ethnicity (NHANES 2011–2016).

Nutrient	Non-Hispanic White			Non-Hispanic Black			Hispanic			Asian		
	Mean ± S.E. ^1^	% < EAR ^2^	% > AI ^2^	Mean ± S.E. ^1^	% < EAR ^2^	% > AI ^2^	Mean ± S.E. ^1^	% < EAR ^2^	% > AI ^2^	Mean ± S.E. ^1^	% < EAR ^2^	% > AI ^2^
Dietary Fiber (g)	11.3 ± 0.2		0.6 ± 0.3	11.9 ± 0.4		0.6 ± 0.3	12 ± 0.2		1.6 ± 0.6	11.2 ± 0.5		1.7 ± 1.3
Vitamin A, RAE (mcg)	592.1 ± 12	1.1 ± 0.7		523.6 ± 16.4	1.8 ± 1.7		560.8 ± 17.4	0.1 ± 0.2		565 ± 23.2	0.3 ± 0.4	
Thiamin (mg)	1.2 ± 0	0 ± 0		1.3 ± 0	0 ± 0		1.3 ± 0	0.1 ± 0.1		1.3 ± 0	0 ± 0	
Riboflavin (mg)	1.7 ± 0	0 ± 0		1.7 ± 0	0 ± 0		1.8 ± 0	0 ± 0		1.8 ± 0.1	0 ± 0	
Niacin (mg)	15.2 ± 0.3	0.1 ± 0.1		17.6 ± 0.4	0 ± 0.1		15.7 ± 0.3	0.2 ± 0.1		15.6 ± 0.8	0.6 ± 0.6	
Vitamin B6 (mg)	1.3 ± 0	0 ± 0		1.5 ± 0	0 ± 0		1.5 ± 0	0 ± 0		1.5 ± 0.1	0 ± 0	
Folate DFE (mcg)	399.7 ± 6.8	0 ± 0 ^a^		415.5 ± 9.9	0.4 ± 0.3 ^ab^		424.4 ± 9.5	0.2 ± 0.1 ^b^		455.9 ± 22.1	0 ± 0.1 ^ab^	
Vitamin B12 (mcg)	4 ± 0.1	0 ± 0		4 ± 0.1	0 ± 0.1		4.3 ± 0.1	0 ± 0		4.6 ± 0.2	0 ± 0	
Vitamin C (mg)	69.5 ± 3.5	2.7 ± 1 ^a^		94.6 ± 2.6	0.4 ± 0.2 ^b^		84.2 ± 3.2	0.1 ± 0.1 ^b^		67.1 ± 5.8	6.8 ± 2.7 ^a^	
Vitamin D (μg)	6.3 ± 0.2	90.4 ± 1.5 ^a^		5.5 ± 0.2	94.9 ± 1.2 ^b^		6.6 ± 0.2	88.5 ± 1.9 ^a^		7.5 ± 0.5	80 ± 5.9 ^a^	
Vitamin E (mg)	5.6 ± 0.1	53.2 ± 3.3 ^a^		6.3 ± 0.2	40.1 ± 3.6 ^b^		5.2 ± 0.1	60.8 ± 2.8 ^a^		5.7 ± 0.3	48.2 ± 9.1 ^a^	
Calcium (mg)	978.6 ± 21	14.3 ± 2.2 ^a^		868.8 ± 21.2	26 ± 3.2 ^b^		994.3 ± 19.7	12.5 ± 2.3 ^a^		958.3 ± 34.8	16.5 ± 3.8 ^ab^	
Iron (mg)	10.7 ± 0.1	1.6 ± 0.3		11.9 ± 0.2	0.9 ± 0.3		11.7 ± 0.3	1.4 ± 0.3		11.5 ± 0.5	0.7 ± 0.7	
Magnesium (mg)	195.4 ± 2.8	0.4 ± 0.2		201.4 ± 4.3	0.4 ± 0.3		198.5 ± 2.7	0.2 ± 0.1		203.5 ± 7.9	0.4 ± 0.3	
Phosphorus (mg)	1075 ± 20	0 ± 0		1054 ± 22	0.1 ± 0.1		1101 ± 16	0 ± 0		1081 ± 40	0 ± 0	
Choline (mg)	202.1 ± 4.2		32.4 ± 2.8 ^a^	218.7 ± 6		42 ± 4.1 ^ab^	229.5 ± 4.2		50.4 ± 3 ^b^	242.3 ± 11.4		54.9 ± 6.9 ^b^
Sodium (mg)	2164 ± 44		99.8 ± 3	2467 ± 61		99.8 ± 0.1	2223 ± 34		99.8 ± 0.1	2178 ± 95		99.7 ± 0.3
Potassium (mg)	1876 ± 34		26.4 ± 2.5 ^a^	2007 ± 40		35.8 ± 3.2 ^b^	2024 ± 27		37.6 ± 2.3 ^ab^	1973 ± 82		33.4 ± 6.9 ^ab^
Zinc (mg)	7.8 ± 0.1	0.1 ± 0.1		8.2 ± 0.2	0.3 ± 0.2		8.2 ± 0.1	0.1 ± 0.1		8.8 ± 0.5	0 ± 0.1	

^1^ Values are means ± S.E. of nutrients from food and beverage intake during a single 24-h dietary recall completed by parents. ^2^ Determined via cut-point method using usual intakes as estimated using the National Cancer Institute method employing use of both dietary recalls compared to recommended intakes. AI: Adequate Intake; DFE: Dietary Folate Equivalent; EAR: Estimated Average Requirement; RAE: Retinol Activity Equivalent. ^ab^ Percentages with different superscripts are significantly different via Z-score, *p* < 0.05.

**Table 4:** Mean nutrient intake of children 1–6 years and proportion not meeting DRIs by Poverty Income Ratio (NHANES 2001–2016).

Nutrient	PIR < 1.3 ^1^			PIR 1.3–1.85 ^1^			PIR ≥ 1.85 ^1^		
	Mean ± S.E. ^2^	% < EAR ^3^	% > AI ^3^	Mean ± S.E. ^2^	% < EAR ^3^	% > AI ^3^	Mean ± S.E. ^2^	% < EAR ^3^	% > AI ^3^
Dietary Fiber (g)	11 ± 0.1		0.7 ± 0.2	11 ± 0.3		1.2 ± 0.4	11 ± 0.2		0.4 ± 0.1
Vitamin A, RAE (mcg)	544.4 ± 7.1	1.8 ± 0.5		556 ± 18.1	0.6 ± 0.6		580.5 ± 9.2	0.9 ± 0.3	
Thiamin (mg)	1.3 ± 0	0 ± 0		1.3 ± 0	0 ± 0.1		1.2 ± 0	0 ± 0	
Riboflavin (mg)	1.9 ± 0	0 ± 0		1.9 ± 0	0 ± 0		1.9 ± 0	0 ± 0	
Niacin (mg)	16.2 ± 0.1	0.2 ± 0		15.9 ± 0.4	0.2 ± 0.1		15.2 ± 0.2	0.1 ± 0	
Vitamin B6 (mg)	1.5 ± 0	0 ± 0		1.4 ± 0	0.1 ± 0.1		1.3 ± 0	0 ± 0	
Folate DFE (mcg)	441.7 ± 6	0.1 ± 0.1		438.2 ± 12	0.1 ± 0.1		416.8 ± 6.4	0 ± 0	
Vitamin B12 (mcg)	4.6 ± 0.1	0 ± 0		4.5 ± 0.1	0 ± 0		4.2 ± 0.1	0.1 ± 0	
Vitamin C (mg)	92.9 ± 2	0.9 ± 0.2		85.2 ± 3.3	1.3 ± 0.6		78.3 ± 2.2	1.4 ± 0.4	
Vitamin D (μg)	7.0 ± 0.1	85 ± 1.1		6.7 ± 0.2	87.4 ± 1.9		6.5 ± 0.1	87.1 ± 1.2	
Vitamin E (mg)	4.9 ± 0.1	67.1 ± 1.5		4.7 ± 0.1	71.5 ± 2.9		4.9 ± 0.1	70.5 ± 1.6	
Calcium (mg)	970.4 ± 13	17.8 ± 1.2		977.1 ± 23.3	16.9 ± 2.4		989.1 ± 13.6	15.8 ± 1	
Iron (mg)	12.1 ± 0.1	1.0 ± 0.2		11.6 ± 0.2	1.1 ± 0.3		11.2 ± 0.1	1.1 ± 0.2	
Magnesium (mg)	198.6 ± 1.9	0.6 ± 0.2		197.6 ± 3.3	0.8 ± 0.3		197.2 ± 2	0.3 ± 0.1	
Phosphorus (mg)	1094 ± 12	0.1 ± 0		1090 ± 20	0.1 ± 0.1		1092 ± 12	0 ± 0	
Choline (mg)	224.1 ± 3.2		46.1 ± 2 ^a^	215.3 ± 6.4		40.6 ± 3.8 ^ab^	206.5 ± 3.1		34.6 ± 1.9 ^b^
Sodium (mg)	2395 ± 25		99.8 ± 0.1	2293 ± 44		99.6 ± 0.3	2263 ± 21		99.9 ± 0.05
Potassium (mg)	2072 ± 21		41.6 ± 1.5	2029 ± 41		39.1 ± 2.7	1978 ± 21		33.4 ± 1.6
Zinc (mg)	8.8 ± 0.1	0.1 ± 0		8.6 ± 0.2	0.3 ± 0.2		8.1 ± 0.1	0.1 ± 0.1	

^1^ <1.3 refers to income less than 1.3 times the poverty level; 1.3–1.85 refers to income 1.3 to 1.85 times the poverty level; > 1.85 refers to income greater than 1.85 times the poverty level. ^2^ Values are means ± S.E. of nutrients from food and beverage intake during two 24-h dietary recalls completed by parents. ^3^ Determined via cut-point method using usual intakes as estimated using the National Cancer Institute method employing use of both dietary recalls compared to recommended intakes. AI: Adequate Intake; DFE: Dietary Folate Equivalent; EAR: Estimated Average Requirement; PIR: Poverty Income Ratio; RAE: Retinol Activity Equivalent. ^ab^ Percentages with different superscripts are significantly different via Z-score, *p* < 0.05.

**Table 5:** Mean and percentile DHA intake of children 1–6 years (NHANES 2001–2016).

Age	Mean ± S.E. ^1^ (mg)	Usual Intake Percentile ^2^					%Population < LL and < UL of Expert Recommendations ^3^
		10	25	50	75	90	
1–2 y	20 ± 1	6	10	16	27	43	97.6–99.3
2–3 y	23 ± 2	6	10	17	28	43	97.6–99.3
1–3 y	22 ± 1	6	10	16	27	43	97.6–99.3
4–6 y	25 ± 2	6	10	17	29	44	97.3–99.2
1–6 y	24 ± 1	6	10	17	28	44	97.4–99.3

^1^ Values are means ± S.E. of DHA intake from food and beverage intake during a single 24-h dietary recall completed by parents. ^2^ Determined with usual intakes as estimated using the National Cancer Institute method employing use of both dietary recalls. ^3^ LL: lower limit 70 mg; UL: upper limit 100 mg, AFFSA [24], EFSA [25].

**Table 6:** Percentage of children 1–6 years with nutrient biomarkers below deficiency levels (NHANES 2001–2016).

Serum Biomarker	Cut Off Value for Deficiency	% Population < Cutoff				
		1–2 y	2–3 y	1–3 y	4–6 y	1–6 y
Ferritin	12 ng/mL	13.0 ± 1.1	9.0 ± 1.0	10.7 ± 1.0 ^a^	3.7 ± 0.7 ^b^	7.4 ± 0.6
Hemoglobin	11 g/dL	4.3 ± 0.5	2.8 ± 0.4	3.6 ± 0.4 ^a^	1.6 ± 0.3 ^b^	2.5 ± 0.3
Folate, RBC	95 ng/mL	0.1 ± 0.1	0.1 ± 0.1	0.1 ± 0.04	0.04 ± 0.04	0.1 ± 0.03
Vitamin B6	20 nmol/L	7.9 ± 1.5	6.4 ± 1.0	7.1 ± 1.1	5.8 ± 1.2	6.4 ± 1.0
Vitamin A	20 μg/dL	NA	1.2 ± 1.0	1.2 ± 1.0	2.0 ± 0.9	1.9 ± 0.8 *
Vitamin B12	200 pg/mL	0.1 ± 0.1	0.0 ± 0.0	0.1 ± 0.1	0.0 ± 0.0	0.0 ± 0.0
Vitamin E	500 μg/dL	NA	NA	NA	0.9 ± 0.8	NA

^ab^ Percentages with different superscripts are significantly different (only the non-overlapping age groups 1–3 and 4–6 y were compared), *p* < 0.05. * Age group is 2–6 y (data not available for those 1 year of age). NA: Not available.

## Data Availability

The NHANES data used in the manuscript and detailed analyses can be found at the following link: https://wwwn.cdc.gov/nchs/nhanes/ContinuousNhanes/Default.aspx (accessed on 1 August 2019).

## Supplementary Materials

The following are available online at https://www.mdpi.com/2072-6643/13/3/827/s1, Table S1: Mean nutrient intake of children 1–6 and proportion not meeting DRIs by Age Group (NHANES 2001–2016); Table S2a: Mean nutrient intake of children 1–2 years and proportion not meeting DRIs by Ethnicity (NHANES 2011–2016); Table S2b: Mean nutrient intake of children 2–3 years and proportion not meeting DRIs by Ethnicity (NHANES 2011–2016); Table S2c: Mean nutrient intake of children 1–3 years and proportion not meeting DRIs by Ethnicity (NHANES 2011–2016); Table S2d: Mean nutrient intake of children 4–6 years and proportion not meeting DRIs by Ethnicity (NHANES 2011–2016); Table S3a: Mean nutrient intake of children 1–2 years and proportion not meeting DRIs by Poverty Income Ratio; Table S3b: Mean nutrient intake of children 2–3 years and proportion not meeting DRIs by Poverty Income Ratio; Table S3c: Mean nutrient intake of children 1–3 years and proportion not meeting DRIs by Poverty Income Ratio; Table S3d: Mean nutrient intake of children 4–6 years and proportion not meeting DRIs by Poverty Income Ratio; Table S4: Logistic Regression of meeting Serum Ferritin, Hemoglobin and Vitamin B6 cutoffs in Children 1–6 Years.
