# Supplementary material for: Nutrient Intake Adequacy from Food and Beverage Intake of US Children Aged 1–6 Years from NHANES 2001–2016
Source: Nutrients. 2021 Mar 3;13(3):827. doi: 10.3390/nu13030827 (PMC8002201; doi:10.3390/nu13030827)
Supplement: Supplementary file 1 [file nutrients-13-00827-s001.pdf]

Table S1. Mean nutrient intake of children 1-6 and proportion not meeting DRIs by Age Group (NHANES 2001-2016)

Nutrient	1-2 y			2-3 y			1-3 y			4-6 y		
	Mean ± S.E. [1]	% < EAR[2]	% > AI[2]	Mean ± S.E. [1]	% < EAR[2]	% > AI[2]	Mean ± S.E. [1]	% < EAR[2]	% > AI[2]	Mean ± S.E. [1]	% < EAR[2]	% > AI[2]
Dietary Fiber (g)	9.5 ± 0.1		0.6 ± 0.1	10.6 ± 0.1		1.4 ± 0.3	9.9 ± 0.1		1.0 ± 0.2 [a]	12.1 ± 0.1		0.2 ± 0.1 [b]
Vitamin A, RAE (µg)	558.7 ± 8.9	0.5 ± 0.1		548.7 ± 8.1	0.5 ± 0.2		552.0 ± 7.1	0.5 ± 0.1 [a]		576.0 ± 8.5	2.1 ± 0.4 [b]	
Thiamin (mg)	1.1 ± 0.0	0.0 ± 0.0		1.2 ± 0.0	0.0 ± 0.0		1.2 ± 0.0	0.0 ± 0.0		1.4 ± 0.0	0.0 ± 0.0	
Riboflavin (mg)	1.9 ± 0.0	0.0 ± 0.0		1.8 ± 0.0	0.0 ± 0.0		1.9 ± 0.0	0.0 ± 0.0		1.9 ± 0.0	0.0 ± 0.0	
Niacin (mg)	12.9 ± 0.1	0.3 ± 0.1		14.5 ± 0.2	0.5 ± 0.0		13.7 ± 0.1	0.2 ± 0.1		17.7 ± 0.2	0.0 ± 0.0	
Vitamin B6 (mg)	1.3 ± 0.0	0.0 ± 0.0		1.35 ± 0.0	0.0 ± 0.0		1.3 ± 0.0	0.0 ± 0.0		1.5 ± 0.0	0.0 ± 0.0	
Folate DFE (µg)	355.5 ± 4.8	0.1 ± 0.1		402.7 ± 6.0	0.0 ± 0.0		376.9 ± 4.6	0.1 ± 0.0		481.3 ± 6.2	0.1 ± 0.0	
Vitamin B12 (µg)	4.3 ± 0.1	0.0 ± 0.0		4.3 ± 0.1	0.0 ± 0.0		4.3 ± 0.1	0.0 ± 0.0		4.5 ± 0.1	0.0 ± 0.0	
Vitamin C (mg)	87.2 ± 2.2	0.3 ± 0.1		89.2 ± 2.0	0.3 ± 0.1		87.4 ± 1.7	0.3 ± 0.1 [a]		82.4 ± 1.9	2.3 ± 0.4 [b]	
Vitamin D (µg)	7.8 ± 0.1	79.2 ± 1.1		6.7 ± 0.1	87.3 ± 1.0		7.3 ± 0.1	82.7 ± 1.0 [a]		6.0 ± 0.1	90.8± 0.8 [b]	
Vitamin E (mg)	4.2 ± 0.1	75.7 ± 1.4		4.6 ± 0.1	65.4 ± 1.5		4.4 ± 0.1	70.9 ± 1.3		5.4 ± 0.1	67.6 ± 1.3	
Calcium (mg)	1018 ± 12	3.0 ± 0.4		973.3 ± 13.5	4.1 ± 0.5		994.3 ± 11.3	3.6 ± 0.4 [a]		962.8 ± 11.3	30.4 ± 1.2 [b]	
Iron (mg)	9.9 ± 0.1	1.2 ± 0.2		10.7 ± 0.1	0.6 ± 0.1		10.3 ± 0.1	0.9 ± 0.1		12.8 ± 0.1	1.3 ± 0.2	
Magnesium (mg)	185.7 ± 1.6	0.0 ± 0.0		193.3 ± 1.8	0.0 ± 0.0		188.5 ± 1.5	0.0 ± 0.0 [a]		207.6 ± 1.7	0.9 ± 0.2 [b]	
Phosphorus (mg)	1051 ± 10	0.1 ± 0.02		1064 ± 11	0.0 ± 0.0		1054 ± 9	0.1 ± 0.0		1130 ± 10	0.0 ± 0.0	
Choline (mg)	209.7 ± 2.4		51.1 ± 1.7	212.1 ± 3.2		52.6 ± 1.8	209.8 ± 2.4		51.7 ± 1.5 [a]	218.1 ± 3		27.1 ± 1.7 [b]
Sodium (mg)	1939 ± 19		99.5 ± 0.1	2176 ± 20		99.9 ± 0.0	2040 ± 16		99.7 ± 0.1 [a]	2591 ± 22		100.0 ± 0.0 [b]
Potassium (mg)	1994 ± 18		44.5 ± 1.5	2007 ± 19		46.8 ± 1.4	1995± 16		45.1 ± 1.3 [a]	2048 ± 21		30.1 ± 1.4 [b]
Zinc (mg)	7.7 ± 0.1	0.0 ± 0.0		8.0 ± 0.0	0.0 ± 0.0		0.0 ± 0.0	0.1 ± 0.1		9.1 ± 0.1	0.3 ± 0.1	

[1]Values are means ± standard error for nutrients from food and beverage intake during two 24-h dietary recalls completed by parents.

[2]Determined via cut-point method using usual intakes as estimated using the National Cancer Institute method employing use of both dietary recalls compared to recommended intakes.

AI: Acceptable Intake; DFE: Dietary Folate Equivalent; EAR: Estimated Average Requirement; RAE: Retinol Activity Equivalent

[ab]Percentages with different superscripts are significantly different using z-scores, $p<0.05$

TABLE S2a. Mean nutrient intake of children 1-2 years and proportion not meeting DRIs by Ethnicity (NHANES 2011-2016)

**Nutrient**	**Non-Hispanic White**			**Non-Hispanic Black**			**Hispanic**			**Asian**		
	**Mean ± S.E.[1]**	**% < EAR[2]**	**% > AI[2]**	**Mean ± S.E.[1]**	**% < EAR[2]**	**% > AI[2]**	**Mean ± S.E.[1]**	**% < EAR[2]**	**% > AI[2]**	**Mean ± S.E.[1]**	**% < EAR[2]**	**% > AI[2]**
Dietary Fiber (g)	10.2 ± 0.3		0.7 ± 0.4	9.8 ± 0.3		0.5 ± 0.3	9.6 ± 0.3		1 ± 0.6	9 ± 0.5		1.3 ± 1
Vitamin A, RAE (µg)	591.8 ± 18	0.4 ± 0.3		523.6 ± 17.4	0.9 ± 1		541.9 ± 16.1	0.02 ± 0.1		532.9 ± 32.7	0.1 ± 0.3	
Thiamin (mg)	1.1 ± 0	0 ± 0		1.1 ± 0	0.1 ± 0.1		1.1 ± 0	0.1 ± 0.1		1.1 ± 0.1	0.0 ± 0.1	
Riboflavin (mg)	1.7 ± 0	0 ± 0		1.6 ± 0	0 ± 0		1.7 ± 0	0 ± 0		1.7 ± 0.1	0 ± 0	
Niacin (mg)	12.9 ± 0.4	0.2 ± 0.2		14.1 ± 0.4	0.2 ± 0.1		12 ± 0.4	0.5 ± 0.4		12 ± 0.8	2.1 ± 1.9	
Vitamin B6 (mg)	1.2 ± 0	0 ± 0		1.3 ± 0	0.0 ± 0.1		1.2 ± 0	0 ± 0		1.2 ± 0.1	0.0 ± 0.2	
Folate DFE (µg)	345.3 ± 13.1	0 ± 0		340.1 ± 13.2	0.8 ± 0.6		347.8 ± 16.2	0.5 ± 0.3		362.2 ± 30.8	0.1 ± 0.2	
Vitamin B12 (µg)	4 ± 0.1	0 ± 0		3.7 ± 0.2	0.0 ± 0.1		4 ± 0.1	0 ± 0		4.3 ± 0.3	0 ± 0	
Vitamin C (mg)	73.2 ± 5.9	0.7 ± 0.4		103.1 ± 5.8	0.0 ± 0.1		78.3 ± 4	0.0 ± 0.1		68.2 ± 9.7	3.4 ± 2.1	
Vitamin D (µg)	7.2 ± 0.3	84.2 ± 2.2 [a]		6.1 ± 0.3	92.7 ± 1.9 [b]		7.7 ± 0.3	80.5 ± 2.8 [a]		8.3 ± 0.5	74 ± 7 [a]	
Vitamin E (mg)	4.8 ± 0.2	58.8 ± 4.1 [a]		5.3 ± 0.2	49.4 ± 4.6 [a]		4.3 ± 0.1	71.1 ± 3.4 [b]		4.7 ± 0.4	65.6 ± 12.2 [ab]	
Calcium (mg)	1021 ± 28	1.3 ± 0.7 [a]		857.0 ± 29	9.7 ± 3 [b]		997.2 ± 26.8	1.6 ± 0.8 [a]		963.6 ± 36.4	2.2 ± 2 [a]	
Iron (mg)	9.4 ± 0.3	1.6 ± 0.4		10.3 ± 0.3	1.0 ± 0.4		9.5 ± 0.4	2.2 ± 0.5		10 ± 1	1.0 ± 1.3	
Magnesium (mg)	184.6 ± 3.9	0 ± 0		180.3 ± 3.7	0.1 ± 0.1		179.6 ± 3.5	0 ± 0		174.6 ± 4.7	0.0 ± 0.1	
Phosphorus (mg)	1037 ± 20	0 ± 0		952.8 ± 24.2	0.2 ± 0.3		1014 ± 24	0 ± 0		967.2 ± 31	0.1 ± 0.1	
Choline (mg)	196.9 ± 5.5		41.6 ± 3.6 [a]	202.8 ± 7.6		47.2 ± 5.4 [a]	224.7 ± 7		63.9 ± 3.4 [b]	216.4 ± 16.9		52.5 ± 11.3 [ab]
Sodium (mg)	1817 ± 46		99.4 ± 0.6	1981 ± 60		99.4 ± 0.4	1757 ± 43		99.5 ± 0.3	1608 ± 88		98.7 ± 1.2
Potassium (mg)	1826 ± 36		29.8 ± 3.2 [a]	1881 ± 39		38.9 ± 3.8 [ab]	1924 ± 39		39 ± 3.3 [b]	1789 ± 63		31.2 ± 7.8 [ab]
Zinc (mg)	7.4 ± 0.2	0 ± 0		7 ± 0.2	0.1 ± 0.1		7 ± 0.2	0.0 ± 0.1		7.2 ± 0.4	0 ± 0	

[1]Values are means ± standard error for nutrients from food and beverage intake during two 24-h dietary recalls completed by parents.

[2]Determined via cut-point method using usual intakes as estimated using the National Cancer Institute method employing use of both dietary recalls compared to recommended intakes.

AI: Acceptable Intake; DFE: Dietary Folate Equivalent; EAR: Estimated Average Requirement; RAE: Retinol Activity Equivalent

[ab]Percentages with different superscripts are significantly different using z-scores, $p<0.05$

TABLE S2b. Mean nutrient intake of children 2-3 years and proportion not meeting DRIs by Ethnicity (NHANES 2011-2016)

Nutrient	Non-Hispanic White			Non-Hispanic Black			Hispanic			Asian		
	Mean ± S.E.[1]	% < EAR[2]	% > AI[2]	Mean ± S.E.[1]	% < EAR[2]	% > AI[2]	Mean ± S.E.[1]	% < EAR[2]	% > AI[2]	Mean ± S.E.[1]	% < EAR[2]	% > AI[2]
Dietary Fiber (g)	10.8 ± 0.4		1.5 ± 0.7	10.8 ± 0.3		1.1 ± 0.6	11.3 ± 0.3		3.4 ± 1.3	10.2 ± 0.9		2.8 ± 3
Vitamin A, RAE (µg)	584.3 ± 17	0.4 ± 0.3		500.6 ± 17.1	1.1 ± 1.2		545.1 ± 28.1	0 ± 0.1		540.5 ± 38.0	00 ± 0.2	
Thiamin (mg)	1.1 ± 0	0 ± 0		1.2 ± 0	0.0 ± 0.1		1.2 ± 0	0 ± 0		1.3 ± 0.1	0 ± 0	
Riboflavin (mg)	1.7 ± 0	0 ± 0		1.7 ± 0	0 ± 0		1.8 ± 0.1	0 ± 0		1.8 ± 0.1	0 ± 0	
Niacin (mg)	14.1 ± 0.4	0.1 ± 0.1		16.5 ± 0.5	0 ± 0		14.5 ± 0.5	0 ± 0		14.9 ± 1.5	0.2 ± 0.5	
Vitamin B6 (mg)	1.3 ± 0	0 ± 0		1.4 ± 0	0 ± 0		1.4 ± 0	0 ± 0		1.5 ± 0.1	0 ± 0	
Folate DFE (µg)	380.3 ± 12.5	0 ± 0		396.7 ± 13.8	0.2 ± 0.2		399 ± 14.3	0.1 ± 0.1		437.2 ± 38	0 ± 0	
Vitamin B12 (µg)	4 ± 0.1	0 ± 0		3.7 ± 0.2	0.0 ± 0.1		4.1 ± 0.2	0 ± 0		4.7 ± 0.5	0 ± 0	
Vitamin C (mg)	72.7 ± 5.5	0.7 ± 0.4		100.1 ± 5	0.1 ± 0.1		86.5 ± 4.9	0 ± 0		69.9 ± 8.6	3.4 ± 2.5	
Vitamin D (µg)	6.4 ± 0.2	90.1 ± 1.4 [a]		5.7 ± 0.2	96.1 ± 1.4 [b]		6.9 ± 0.3	88.3 ± 2.5 [a]		8.4 ± 1.2	76.1 ± 10.8 [ab]	
Vitamin E (mg)	5.2 ± 0.2	48.6 ± 4.5		5.9 ± 0.3	40.9 ± 5.3		5.2 ± 0.2	50.5 ± 4.2		5.3 ± 0.4	48.5 ± 10	
Calcium (mg)	983.2 ± 33	1.9 ± 0.9 [a]		839.1 ± 25.2	11.9 ± 3.2 [b]		1020.2 ± 45.5	1.6 ± 1.1 [a]		952.8 ± 72.8	2.1 ± 1.5 [a]	
Iron (mg)	9.8 ± 0.3	1.1 ± 0.3		10.9 ± 0.2	0.5 ± 0.2		10.8 ± 0.4	0.6 ± 0.2		10.8 ± 0.7	0.3 ± 0.4	
Magnesium (mg)	190.6 ± 4.8	0 ± 0		194.6 ± 4.7	0 ± 0		194.9 ± 5.4	0 ± 0		196.5 ± 15.5	0 ± 0	
Phosphorus (mg)	1061 ± 26	0 ± 0		1008 ± 26	0.1 ± 0.2		1084 ± 36	0 ± 0		1076 ± 83	0 ± 0	
Choline (mg)	204.6 ± 7.5		45.3 ± 4.4 [a]	216 ± 6.5		51.5 ± 4.7 [a]	231.9 ± 7.9		70.8 ± 4.3 [b]	232.7 ± 20.7		66.5 ± 10.3 [ab]
Sodium (mg)	2031 ± 51		99.9 ± 0.1	2259 ± 64		99.9 ± 0.1	2129 ± 67		100 ± 0.1	1992 ± 147		99.9 ± 0.2
Potassium (mg)	1877 ± 49		35.9 ± 4.1	1968 ± 53		41.6 ± 4.7	2009 ± 61		47.1 ± 5	1967 ± 192		44.2 ± 11.4
Zinc (mg)	7.6 ± 0.2	0 ± 0		7.6 ± 0.2	0.1 ± 0.1		7.8 ± 0.3	0 ± 0		7.6 ± 0.6	0 ± 0	

[1]Values are means ± standard error for nutrients from food and beverage intake during two 24-h dietary recalls completed by parents.

[2]Determined via cut-point method using usual intakes as estimated using the National Cancer Institute method employing use of both dietary recalls compared to recommended intakes.

AI: Acceptable Intake; DFE: Dietary Folate Equivalent; EAR: Estimated Average Requirement; RAE: Retinol Activity Equivalent

[ab]Percentages with different superscripts are significantly different using z-scores, $p<0.05$

TABLE S2c. Mean nutrient intake of children 1-3 years and proportion not meeting DRIs by Ethnicity (NHANES 2011-2016)

Nutrient	Non-Hispanic White			Non-Hispanic Black			Hispanic			Asian		
	**Mean ± S.E.[1]**	**% < EAR[2]**	**% > AI[2]**	**Mean ± S.E.[1]**	**% < EAR[2]**	**% > AI[2]**	**Mean ± S.E.[1]**	**% < EAR[2]**	**% > AI[2]**	**Mean ± S.E.[1]**	**% < EAR[2]**	**% > AI[2]**
Dietary Fiber (g)	10.4 ± 0.3		1.1 ± 0.5	10.3 ± 0.3		0.9 ± 0.5	10.4 ± 0.3		2.5 ± 0.9	9.6 ± 0.7		2.3 ± 2.1
Vitamin A, RAE [illegible]	584.6 ± 13.8	0.4 ± 0.3		510.9 ± 12.9	0.9 ± 1.1		545.4 ± 21.1	0 ± 0.1		543.5 ± 27.8	0 ± 0.2	
Thiamin (mg)	1.1 ± 0	0 ± 0		1.2 ± 0	0.1 ± 0.1		1.1 ± 0	0.1 ± 0.1		1.2 ± 0.1	0 ± 0.1	
Riboflavin (mg)	1.7 ± 0	0 ± 0		1.6 ± 0	0 ± 0		1.7 ± 0	0 ± 0		1.8 ± 0.1	0 ± 0	
Niacin (mg)	13.6 ± 0.3	0.2 ± 0.1		15.3 ± 0.5	0.1 ± 0.1		13.2 ± 0.3	0.3 ± 0.2		13.7 ± 1	1.3 ± 1.1	
Vitamin B6 (mg)	1.2 ± 0	0 ± 0		1.3 ± 0	0 ± 0		1.3 ± 0	0 ± 0		1.4 ± 0.1	0.1 ± 0.1	
Folate DFE (µg)	364.9 ± 10.1	0 ± 0 [a]		367.1 ± 12.3	0.6 ± 0.4 [ab]		368.3 ± 11.1	0.4 ± 0.2 [b]		412 ± 25.9	0 ± 0.1 [ab]	
Vitamin B12 (µg)	4 ± 0.1	0 ± 0		3.7 ± 0.1	0 ± 0		4.1 ± 0.1	0 ± 0		4.6 ± 0.3	0 ± 0	
Vitamin C (mg)	71.7 ± 4	0.6 ± 0.3 [a]		100.6 ± 4.3	0.1 ± 0.1 [ab]		82 ± 3.6	0 ± 0 [b]		70.2 ± 7	3.1 ± 1.7 [ab]	
Vitamin D (µg)	6.9 ± 0.2	86.6 ± 1.7 [a]		6 ± 0.2	93.9 ± 1.6 [b]		7.3 ± 0.3	84.1 ± 2.5 [a]		8.6 ± 0.8	72.1 ± 8.8 [a]	
Vitamin E (mg)	5 ± 0.2	53 ± 4.1 [ab]		5.7 ± 0.2	43.8 ± 4.9 [a]		4.7 ± 0.1	60.6 ± 2.9 [b]		5.1 ± 0.3	54.8 ± 7.5 [ab]	
Calcium (mg)	997.9 ± 27.5	1.7 ± 0.9 [a]		856.8 ± 21.3	10.2 ± 2.8 [b]		1003 ± 30	1.5 ± 0.9 [a]		974.9 ± 51.8	1.8 ± 1.5 [a]	
Iron (mg)	9.7 ± 0.2	1.1 ± 0.3		10.5 ± 0.2	0.7 ± 0.3		10.1 ± 0.3	1.5 ± 0.4		10.7 ± 0.7	0.5 ± 0.7	
Magnesium (mg)	186.9 ± 3.9	0 ± 0		188.6 ± 4.1	0.0 ± 0.1		186.2 ± 3.6	0 ± 0		188.4 ± 11	0 ± 0.1	
Phosphorus (mg)	1045 ± 20	0 ± 0		986.7 ± 21.6	0.1 ± 0.2		1046 ± 24	0 ± 0		1036 ± 59	0 ± 0.1	
Choline (mg)	203.1 ± 5.8		44.8 ± 3.8 [a]	211.5 ± 5.6		51.5 ± 4.3 [a]	226.1 ± 5.7		66.7 ± 3.2 [b]	224.6 ± 16.7		62.9 ± 9.1 [ab]
Sodium (mg)	1922 ± 45		99.6 ± 0.4	2117 ± 58		99.6 ± 0.2	1934 ± 47		99.7 ± 0.2	1825 ± 116		99.4 ± 0.7
Potassium (mg)	1856 ± 39		33.4 ± 3.6 [a]	1928 ± 43		41.2 ± 4 [ab]	1955 ± 40		43.2 ± 3.4 [b]	1907 ± 139		39.8 ± 8.9 [ab]
Zinc (mg)	7.6 ± 0.1	0 ± 0		7.3 ± 0.2	0.1 ± 0.1		7.3 ± 0.2	0 ± 0		7.5 ± 0.4	0 ± 0	

[1]Values are means ± standard error for nutrients from food and beverage intake during two 24-h dietary recalls completed by parents.

[2]Determined via cut-point method using usual intakes as estimated using the National Cancer Institute method employing use of both dietary recalls compared to recommended intakes.

AI: Acceptable Intake; DFE: Dietary Folate Equivalent; EAR: Estimated Average Requirement; RAE: Retinol Activity Equivalent

[ab]Percentages with different superscripts are significantly different using z-scores, p<0.05

TABLE S2d. Mean nutrient intake of children 4-6 years and proportion not meeting DRIs by Ethnicity (NHANES 2011-2016)

Nutrient	Non-Hispanic White			Non-Hispanic Black			Hispanic			Asian		
	Mean ± S.E.[1]	% < EAR[2]	% > AI[2]	Mean ± S.E.[1]	% < EAR[2]	% > AI[2]	Mean ± S.E.[1]	% < EAR[2]	% > AI[2]	Mean ± S.E.[1]	% < EAR[2]	% > AI[2]
Dietary Fiber (g)	12.3 ± 0.3		0.1 ± 0.1	13.3 ± 0.5		0.2 ± 0.2	13.7 ± 0.4		0.7 ± 0.4	12.6 ± 0.7		0.9 ± 0.9
Vitamin A, RAE (µg)	600.0 ± 21.2	1.8 ± 1.1		535.1 ± 25.9	2.5 ± 2.3		576.9 ± 22.7	0.2 ± 0.4		584.0 ± 34.1	0.5 ± 0.8	
Thiamin (mg)	1.3 ± 0	0 ± 0		1.4 ± 0	0 ± 0		1.4 ± 0	0 ± 0		1.4 ± 0.1	0 ± 0	
Riboflavin (mg)	1.7 ± 0	0 ± 0		1.8 ± 0.1	0 ± 0		1.8 ± 0.1	0 ± 0		1.8 ± 0.1	0 ± 0	
Niacin (mg)	16.8 ± 0.5	0.0 ± 0.1		19.8 ± 0.5	0 ± 0		18.4 ± 0.5	0 ± 0		17.2 ± 0.9	0.1 ± 0.2	
Vitamin B6 (mg)	1.3 ± 0	0 ± 0		1.6 ± 0	0 ± 0		1.6 ± 0	0 ± 0		1.6 ± 0.1	0 ± 0	
Folate DFE (µg)	436.6 ± 12.6	0.0 ± 0.1		459.3 ± 13.6	0.2 ± 0.2		483.4 ± 14.6	0.1 ± 0.1		494.4 ± 31.3	0 ± 0.1	
Vitamin B12 (µg)	3.9 ± 0.1	0.0 ± 0.1		4.2 ± 0.2	0.1 ± 0.1		4.5 ± 0.2	0 ± 0		4.6 ± 0.4	0 ± 0	
Vitamin C (mg)	67.1 ± 5.6	4.9 ± 1.9 [a]		89.1 ± 2.9	0.7 ± 0.3 [b]		86.5 ± 4.4	0.3 ± 0.2 [b]		64.3 ± 6.9	9.4 ± 3.8 [a]	
Vitamin D (µg)	5.6 ± 0.3	94.3 ± 1.7 [ab]		5.1 ± 0.2	96.1 ± 1.2 [a]		6 ± 0.3	93 ± 1.7 [ab]		6.5 ± 0.4	86.5 ± 4.6 [b]	
Vitamin E (mg)	6.1 ± 0.2	53.1 ± 4.1 [a]		6.9 ± 0.2	36.5 ± 3.6 [b]		5.8 ± 0.2	60.5 ± 3.7 [a]		6.2 ± 0.4	42.2 ± 12 [ab]	
Calcium (mg)	958.1 ± 31.2	27.9 ± 4 [a]		879.7 ± 28.8	40.7 ± 4.5 [b]		985.8 ± 29.9	24.2 ± 4 [a]		943.7 ± 45.8	28.2 ± 6.1 [a]	
Iron (mg)	11.7 ± 0.2	2.1 ± 0.3 [a]		13.2 ± 0.4	1.0 ± 0.4 [a]		13.4 ± 0.4	1.3 ± 0.3 [ab]		12.2 ± 0.6	0.8 ± 0.8 [ab]	
Magnesium (mg)	204.4 ± 4.8	0.7 ± 0.4		212.8 ± 6.1	0.8 ± 0.5		211.5 ± 4	0.4 ± 0.2		216.8 ± 9.8	0.7 ± 0.6	
Phosphorus (mg)	1107 ± 30	0 ± 0		1116 ± 30	0.0 ± 0.1		1158 ± 24	0 ± 0		1121 ± 45	0 ± 0	
Choline (mg)	201 ± 6.2		19.1 ± 3.3 [a]	225.2 ± 9.2		33.4 ± 4.9 [b]	233.1 ± 6.6		33 ± 4.1 [b]	257.8 ± 15.1		47.8 ± 6.6 [b]
Sodium (mg)	2421 ± 69		99.9 ± 0.1	2782 ± 83		99.9 ± 0.1	2526 ± 48		100.0 ± 0.0	2488 ± 116		100.0 ± 0.1
Potassium (mg)	1896 ± 55		18.4 ± 3.2 [a]	2078 ± 53		31.2 ± 3.4 [b]	2097 ± 41		31.7 ± 2.7 [b]	2031 ± 85		28.9 ± 7 [ab]
Zinc (mg)	8.1 ± 0.2	0.3 ± 0.2		9.1 ± 0.3	0.6 ± 0.4		9 ± 0.2	0.2 ± 0.2		9.9 ± 0.8	0.0 ± 0.1	

[1]Values are means ± standard error for nutrients from food and beverage intake during two 24-h dietary recalls completed by parents.

[2]Determined via cut-point method using usual intakes as estimated using the National Cancer Institute method employing use of both dietary recalls compared to recommended intakes.

AI: Acceptable Intake; DFE: Dietary Folate Equivalent; EAR: Estimated Average Requirement; RAE: Retinol Activity Equivalent

[ab]Percentages with different superscripts are significantly different using z-scores, $p<0.05$

TABLE S3a. Mean nutrient intake of children 1-2 years and proportion not meeting DRIs by Poverty Income Ratio

Nutrient	PIR < 1.3[1]			PIR 1.3-1.85[1]			PIR ≥ 1.85[1]		
	**Mean ± S.E.[2]**	**% < EAR[3]**	**% > AI[3]**	**Mean ± S.E.[2]**	**% < EAR[3]**	**% > AI[3]**	**Mean ± S.E.[2]**	**% < EAR[3]**	**% > AI[3]**
Dietary Fiber (g)	9.2 ± 0.1		0.8 ± 0.2	9.5 ± 0.4		1.2 ± 0.5	9.6 ± 0.2		0.4 ± 0.2
Vitamin A, RAE (µg)	534.4 ± 9.5	0.8 ± 0.3		560.6 ± 31.7	0.2 ± 0.3		577.9 ± 12.5	0.3 ± 0.2	
Thiamin (mg)	1.2 ± 0.0	0.1 ± 0.0		1.1 ± 0	0.1 ± 0.1		1.1 ± 0	0 ± 0	
Riboflavin (mg)	1.9 ± 0.0	0 ± 0		1.9 ± 0.1	0 ± 0		1.9 ± 0	0 ± 0	
Niacin (mg)	13.5 ± 0.2	0.4 ± 0.1		13.1 ± 0.4	0.5 ± 0.3		12.4 ± 0.2	0.2 ± 0.1	
Vitamin B6 (mg)	1.3 ± 0.0	0 ± 0		1.3 ± 0	0.1 ± 0.1		1.2 ± 0	0 ± 0	
Folate DFE (µg)	369.8 ± 7.4	0.3 ± 0.1 [a]		373.5 ± 15.2	0.2 ± 0.2 [ab]		339.4 ± 6.7	0.1 ± 0.1 [b]	
Vitamin B12 (µg)	4.5 ± 0.1	0 ± 0		4.5 ± 0.3	0 ± 0		4.2 ± 0.1	0 ± 0	
Vitamin C (mg)	99.3 ± 3.2	0.2 ± 0.1		90.2 ± 6.1	0.2 ± 0.2		76.3 ± 3.1	0.5 ± 0.2	
Vitamin D (µg)	7.8 ± 0.1	78.5 ± 1.4		7.7 ± 0.3	81.8 ± 2.7		7.7 ± 0.2	78.9 ± 1.7	
Vitamin E (mg)	4.3 ± 0.1	72.8 ± 1.9		4.1 ± 0.2	76.1 ± 3.6		4.1 ± 0.1	77.5 ± 2.1	
Calcium (mg)	998 ± 15.8	4.0 ± 0.6 [a]		1012 ± 32	3.3 ± 1.3 [ab]		1037 ± 19	2.1 ± 0.5 [b]	
Iron (mg)	10.4 ± 0.2	1.2 ± 0.2		10.3 ± 0.4	0.9 ± 0.4		9.5 ± 0.2	1.3 ± 0.2	
Magnesium (mg)	185.6 ± 2.2	0.1 ± 0.0		186.6 ± 5.9	0.1 ± 0.1		185.4 ± 2.4	0 ± 0	
Phosphorus (mg)	1042 ± 13	0.1 ± 0.0		1052 ± 28	0.1 ± 0.1		1059 ± 16	0 ± 0	
Choline (mg)	221 ± 4.2		57.4 ± 2.7 [a]	206.5 ± 6.3		47.3 ± 4.5 [ab]	201.4 ± 3.3		46.8 ± 2.4 [b]
Sodium (mg)	2022 ± 28		99.5 ± 0.2	1943 ± 55		98.9 ± 0.7	1869 ± 30		99.8 ± 0.1
Potassium (mg)	2040 ± 25		49 ± 1.8 [a]	1975 ± 59		42.7 ± 3.9	1958 ± 26		40.8 ± 2.2 [b]
Zinc (mg)	7.9 ± 0.1	0 ± 0		7.9 ± 0.2	0.1 ± 0.1		7.4 ± 0.1	0 ± 0	

[1] PIR< 1.3 refers to income less than 1.3 times the poverty level; PIR 1.3 – 1.85 refers to income 1.3 to 1.85 times the poverty level; PIR > 1.85 refers to income greater than 1.85 times the poverty level.

[2]Values are means ± standard error for nutrients from food and beverage intake during two 24-h dietary recalls completed by parents.

[3]Determined via cut-point method using usual intakes as estimated using the National Cancer Institute method employing use of both dietary recalls compared to recommended intakes.

AI: Acceptable Intake; DFE: Dietary Folate Equivalent; EAR: Estimated Average Requirement; PIR: Poverty Income Ratio; RAE: Retinol Activity Equivalent

PIR< 1.3 refers to income less than 1.3 times the poverty level; PIR 1.3 – 1.85 refers to income 1.3 to 1.85 times the poverty level; PIR > 1.85 refers to income greater than 1.85 times the poverty level.

[ab]Percentages with different superscripts are significantly different using z-scores, $p<0.05$

TABLE S3b. Mean nutrient intake of children 2-3 years and proportion not meeting DRIs by Poverty Income Ratio

Nutrient	PIR < 1.3[1]			PIR 1.3-1.85[1]			PIR ≥ 1.85[1]		
	Mean ± S.E.[2]	% < EAR[3]	% > AI[3]	Mean ± S.E.[1]	% < EAR[2]	Mean ± S.E.[2]	Mean ± S.E.[2]	% < EAR[3]	% > AI[3]
Dietary Fiber (g)	10.4 ± 0.2		1.7 ± 0.4	10.9 ± 0.4		2.7 ± 1	10.6 ± 0.2		0.9 ± 0.3
Vitamin A, RAE (µg)	518 ± 10.3	1.0 ± 0.3		553.3 ± 29.4	0.2 ± 0.3		569.3 ± 12.3	0.3 ± 0.2	
Thiamin (mg)	1.2 ± 0	0 ± 0		1.2 ± 0	0 ± 0		1.1 ± 0	0 ± 0	
Riboflavin (mg)	1.8 ± 0	0 ± 0		1.9 ± 0	0 ± 0		1.8 ± 0	0 ± 0	
Niacin (mg)	15.2 ± 0.2	0 ± 0		15 ± 0.5	0.2 ± 0.2		13.7 ± 0.2	0.1 ± 0.0	
Vitamin B6 (mg)	1.4 ± 0	0 ± 0		1.4 ± 0	0 ± 0		1.3 ± 0	0 ± 0	
Folate DFE (µg)	422.2 ± 10.3	0 ± 0		425.9 ± 19.2	0 ± 0		379.2 ± 9.3	0 ± 0	
Vitamin B12 (µg)	4.5 ± 0.1	0 ± 0		4.6 ± 0.3	0 ± 0		4.1 ± 0.1	0 ± 0	
Vitamin C (mg)	99.4 ± 3	0.2 ± 0.1		96.0 ± 5.6	0.2 ± 0.2		80.1 ± 3.3	0.4 ± 0.2	
Vitamin D (µg)	6.8 ± 0.2	86 ± 1.3		6.8 ± 0.3	88.8 ± 2.2		6.6 ± 0.2	87.2 ± 1.4	
Vitamin E (mg)	4.6 ± 0.1	62.5 ± 2		4.4 ± 0.1	68.1 ± 3.4		4.5 ± 0.1	67.6 ± 2.6	
Calcium (mg)	944 ± 18.9	5.4 ± 0.9 [a]		989.4 ± 40.2	4.3 ± 1.7 [ab]		990.5 ± 20.2	2.7 ± 0.6 [b]	
Iron (mg)	11.3 ± 0.2	0.4 ± 0.1 [a]		11.1 ± 0.3	0.5 ± 0.2 [ab]		10.1 ± 0.2	0.7 ± 0.1 [b]	
Magnesium (mg)	192 ± 2.8	0 ± 0		199.3 ± 5.2	0 ± 0		192.2 ± 3.1	0 ± 0	
Phosphorus (mg)	1054 ± 16	0 ± 0		1082 ± 26	0.1 ± 0.1		1066 ± 17	0 ± 0	
Choline (mg)	221 ± 4.4		59.5 ± 2.5 [a]	218 ± 12.7		57.8 ± 6.7 [ab]	205.3 ± 4.2		47.6 ± 2.8 [b]
Sodium (mg)	2272 ± 33		99.9 ± 0	2223 ± 63		99.8 ± 0.2	2085 ± 31		100.0 ± 0
Potassium (mg)	2034 ± 34		49.2 ± 2.3	2078 ± 54		50.4 ± 3.4	1961 ± 29		43.2 ± 2.2
Zinc (mg)	8.4 ± 0.2	0 ± 0		8.4 ± 0.3	0 ± 0		7.6 ± 0.1	0 ± 0	

[1] PIR< 1.3 refers to income less than 1.3 times the poverty level; PIR 1.3 – 1.85 refers to income 1.3 to 1.85 times the poverty level; PIR > 1.85 refers to income greater than 1.85 times the poverty level.

[2]Values are means ± standard error for nutrients from food and beverage intake during two 24-h dietary recalls completed by parents.

[3]Determined via cut-point method using usual intakes as estimated using the National Cancer Institute method employing use of both dietary recalls compared to recommended intakes.

AI: Acceptable Intake; DFE: Dietary Folate Equivalent; EAR: Estimated Average Requirement; PIR: Poverty Income Ratio; RAE: Retinol Activity Equivalent

PIR< 1.3 refers to income less than 1.3 times the poverty level; PIR 1.3 – 1.85 refers to income 1.3 to 1.85 times the poverty level; PIR > 1.85 refers to income greater than 1.85 times the poverty level.

[ab]Percentages with different superscripts are significantly different using z-scores, $p<0.05$

TABLE S3c. Mean nutrient intake of children 1-3 years and proportion not meeting DRIs by Poverty Income Ratio

Nutrient	PIR < 1.3[1]			PIR 1.3-1.85[1]			PIR ≥ 1.85[1]		
	**Mean ± S.E.[2]**	**% < EAR[3]**	**% > AI[3]**	**Mean ± S.E.[2]**	**% < EAR[3]**	**% > AI[3]**	**Mean ± S.E.[2]**	**% < EAR[3]**	**% > AI[3]**
Dietary Fiber (g)	9.7 ± 0.1		1.2 ± 0.3	10.2 ± 0.3		2.1 ± 0.7	10 ± 0.2		0.7 ± 0.2
Vitamin A, RAE (µg)	525.1 ± 8.4	0.9 ± 0.3		543.5 ± 23.1	0.2 ± 0.3		573.7 ± 10.3	0.3 ± 0.2	
Thiamin (mg)	1.2 ± 0	0 ± 0		1.2 ± 0	0.1 ± 0.1		1.1 ± 0	0 ± 0	
Riboflavin (mg)	1.9 ± 0	0 ± 0		1.9 ± 0	0 ± 0		1.8 ± 0	0 ± 0	
Niacin (mg)	14.1 ± 0.2	0.3 ± 0.1		14.1 ± 0.4	0.3 ± 0.2		13.1 ± 0.2	0.2 ± 0.1	
Vitamin B6 (mg)	1.4 ± 0	0 ± 0		1.3 ± 0	0.1 ± 0.1		1.2 ± 0	0 ± 0	
Folate DFE (µg)	389.8 ± 7.5	0.2 ± 0.1 [a]		398.5 ± 14.8	0.1 ± 0.1 [ab]		359.4 ± 7.1	0 ± 0 [b]	
Vitamin B12 (µg)	4.5 ± 0.1	0 ± 0		4.4 ± 0.2	0 ± 0		4.1 ± 0.1	0 ± 0	
Vitamin C (mg)	97.3 ± 2.6	0.2 ± 0.1		92.1 ± 4.8	0.2 ± 0.2		78.1 ± 2.7	0.4 ± 0.2	
Vitamin D (µg)	7.4 ± 0.1	81.7 ± 1.2		7.1 ± 0.2	85.1 ± 2.1		7.2 ± 0.1	82.5 ± 1.5	
Vitamin E (mg)	4.4 ± 0.1	68.8 ± 1.7		4.3 ± 0.1	70.8 ± 3		4.3 ± 0.1	72.6 ± 2.2	
Calcium (mg)	974 ± 15.2	4.8 ± 0.8 [a]		993.3 ± 31.6	4 ± 1.5 [ab]		1012 ± 17	2.5 ± 0.5 [b]	
Iron (mg)	10.8 ± 0.2	0.8 ± 0.2		10.8 ± 0.3	0.6 ± 0.2		9.9 ± 0.2	1.0 ± 0.2	
Magnesium (mg)	187.8 ± 2.2	0 ± 0		191.1 ± 4.2	0.1 ± 0.1		187.6 ± 2.5	0 ± 0	
Phosphorus (mg)	1046 ± 13	0.1 ± 0.0		1061 ± 21	0.1 ± 0.1		1058 ± 14	0 ± 0	
Choline (mg)	220 ± 3.8		57.9 ± 2.4 [a]	213.3 ± 9		53.5 ± 4.7 [ab]	202.2 ± 3.1		46.1 ± 2.3 [b]
Sodium (mg)	2122 ± 26		99.6 ± 0.1	2082 ± 53		99.4 ± 0.5	1965 ± 27		99.8 ± 0.1
Potassium (mg)	2030 ± 26		48.4 ± 1.8	2016 ± 43		46.9 ± 2.8	1955 ± 24		41.7 ± 2.1
Zinc (mg)	8.1 ± 0.1	0 ± 0		8.1 ± 0.2	0.1 ± 0.1		7.5 ± 0.1	0 ± 0	

[1] PIR< 1.3 refers to income less than 1.3 times the poverty level; PIR 1.3 – 1.85 refers to income 1.3 to 1.85 times the poverty level; PIR > 1.85 refers to income greater than 1.85 times the poverty level.

[2]Values are means ± standard error for nutrients from food and beverage intake during two 24-h dietary recalls completed by parents.

[3]Determined via cut-point method using usual intakes as estimated using the National Cancer Institute method employing use of both dietary recalls compared to recommended intakes.

AI: Acceptable Intake; DFE: Dietary Folate Equivalent; EAR: Estimated Average Requirement; PIR: Poverty Income Ratio; RAE: Retinol Activity Equivalent

PIR< 1.3 refers to income less than 1.3 times the poverty level; PIR 1.3 – 1.85 refers to income 1.3 to 1.85 times the poverty level; PIR > 1.85 refers to income greater than 1.85 times the poverty level.

[ab]Percentages with different superscripts are significantly different using z-scores, $p<0.05$

TABLE S3d. Mean nutrient intake of children 4-6 years and proportion not meeting DRIs by Poverty Income Ratio

Nutrient	PIR < 1.3[1]			PIR 1.3-1.85[1]			PIR ≥ 1.85[1]		
	Mean ± S.E.[2]	% < EAR[3]	% > AI[3]	Mean ± S.E.[2]	% < EAR[3]	% > AI[3]	Mean ± S.E.[2]	% < EAR[3]	% > AI[3]
Dietary Fiber (g)	12.4 ± 0.2		0.3 ± 0.1	11.8 ± 0.3		0.3 ± 0.2	12 ± 0.2		0.1 ± 0.1
Vitamin A, RAE (µg)	565.1 ± 11.3	2.8 ± 0.7		569.2 ± 23.5	1.1 ± 0.9		587.1 ± 13.5	1.5 ± 0.5	
Thiamin (mg)	1.4 ± 0	0 ± 0		1.4 ± 0	0.0 ± 0.1		1.4 ± 0	0 ± 0	
Riboflavin (mg)	2 ± 0	0 ± 0		1.9 ± 0.1	0 ± 0		1.9 ± 0	0 ± 0	
Niacin (mg)	18.4 ± 0.2	0 ± 0		17.7 ± 0.5	0.0 ± 0.1		17.3 ± 0.3	0 ± 0	
Vitamin B6 (mg)	1.6 ± 0	0 ± 0		1.6 ± 0.1	0.1 ± 0.1		1.4 ± 0	0 ± 0	
Folate DFE (µg)	497.5 ± 9.4	0.1 ± 0.1		480.2 ± 18.9	0.1 ± 0.1		472.6 ± 9.9	0 ± 0	
Vitamin B12 (µg)	4.8 ± 0.1	0 ± 0		4.6 ± 0.1	0 ± 0		4.2 ± 0.1	0.1 ± 0.0	
Vitamin C (mg)	88.2 ± 2.4	1.7 ± 0.4		78 ± 4.3	2.4 ± 1.1		78.5 ± 2.8	2.3 ± 0.6	
Vitamin D (µg)	6.4 ± 0.2	88.8 ± 1.3		6.2 ± 0.2	89.8 ± 2.2		5.7 ± 0.2	91.8 ± 1.2	
Vitamin E (mg)	5.4 ± 0.1	65.1 ± 2.2		5.2 ± 0.2	71.3 ± 3.7		5.4 ± 0.1	68.5 ± 1.9	
Calcium (mg)	966.5 ± 19.9	31.9 ± 2.1		960.1 ± 34.8	30.9 ± 3.9		967.2 ± 18.2	28.5 ± 1.8	
Iron (mg)	13.5 ± 0.2	1.1 ± 0.2		12.4 ± 0.3	1.6 ± 0.4		12.4 ± 0.2	1.3 ± 0.2	
Magnesium (mg)	210.1 ± 3	1.2 ± 0.3[ab]		204.5 ± 4.4	1.7 ± 0.5[a]		206.5 ± 2.8	0.5 ± 0.2[b]	
Phosphorus (mg)	1146 ± 18	0 ± 0		1121 ± 30	0.1 ± 0.1		1125 ± 16	0 ± 0	
Choline (mg)	228.1 ± 4.5		34.3 ± 2.4[a]	217.2 ± 6.9		27.2 ± 4.3[ab]	210.8 ± 4.7		22.5 ± 2.9[b]
Sodium (mg)	2689 ± 37		99.9 ± 0.0[ab]	2515 ± 65		99.8 ± 0.1[a]	2554 ± 33		100.0 ± 0.0[b]
Potassium (mg)	2117 ± 32		34.7 ± 1.9[a]	2043 ± 59		31.0 ± 3.6[ab]	2001 ± 30		25.7 ± 2.0[b]
Zinc (mg)	9.6 ± 0.1	0.2 ± 0.1		9.1 ± 0.3	0.7 ± 0.4		8.8 ± 0.2	0.2 ± 0.1	

[1] PIR< 1.3 refers to income less than 1.3 times the poverty level; PIR 1.3 – 1.85 refers to income 1.3 to 1.85 times the poverty level; PIR > 1.85 refers to income greater than 1.85 times the poverty level.

[2]Values are means ± standard error for nutrients from food and beverage intake during two 24-h dietary recalls completed by parents.

[3]Determined via cut-point method using usual intakes as estimated using the National Cancer Institute method employing use of both dietary recalls compared to recommended intakes.

AI: Acceptable Intake; DFE: Dietary Folate Equivalent; EAR: Estimated Average Requirement; PIR: Poverty Income Ratio; RAE: Retinol Activity Equivalent

PIR< 1.3 refers to income less than 1.3 times the poverty level; PIR 1.3 – 1.85 refers to income 1.3 to 1.85 times the poverty level; PIR > 1.85 refers to income greater than 1.85 times the poverty level.

[ab]Percentages with different superscripts are significantly different using z-scores, $p<0.05$

Table 4S. Logistic Regression of meeting Serum Ferritin, Hemoglobin and Vitamin B6 cutoffs in Children 1-6 Years

Biomarker	NHANES	N	Events	Variable	OR	LCL99-UCL99	P
Ferritin < 12 mg/dL	2011-2016	544	20	Male	1.15	0.46-2.88	0.686
				Hispanic	5.54	0.91-33.57	**0.015**
				Non-Hispanic Black	0.58	0.02-16.98	0.676
				Asian	13.58	0.53-348.15	**0.038**
				Other	0.00	0.00-0.00	**<0.001**
				WIC Eligible = Yes	0.52	0.17-1.62	0.136
				WIC Benefits = Yes	2.63	0.92-7.51	**0.018**
	2001-2016	3,942	332	Male	1.37	0.89-2.10	0.058
				Mexican American	1.03	0.66-1.60	0.876
				Other Hispanic	0.82	0.42-1.62	0.460
				Non-Hispanic Black	0.48	0.25-0.90	**0.003**
				Other	0.73	0.27-1.96	0.414
				WIC Eligible = Yes	0.84	0.52-1.35	0.334
				WIC Benefits = Yes	1.52	0.94-2.47	**0.025**
Hemoglobin < 11 mg/dL	2001-2016	6,802	215	Male	1.07	0.67-1.71	0.713
				Mexican American	1.32	0.66-2.63	0.304
				Other Hispanic	1.50	0.63-3.59	0.229
				Non-Hispanic Black	3.28	1.74-6.21	**<0.001**
				Other	1.06	0.31-3.58	0.899
				WIC Eligible = Yes	1.03	0.59-1.78	0.905
				WIC Benefits = Yes	1.41	0.82-2.41	0.099
Vitamin B6: Pyridoxal-5-phosphate < 20 nmol/L	2001-2016	3,177	208	Male	1.12	0.64-1.97	0.592
				Mexican American	0.62	0.16-2.46	0.373
				Other Hispanic	0.48	0.09-2.55	0.261
				Non-Hispanic Black	0.75	0.29-1.93	0.441
				Other	0.61	0.16-2.34	0.347
				WIC Eligible = Yes	1.96	0.89-4.33	**0.029**
				WIC Benefits = Yes	1.00	0.58-1.73	0.981

OR: odds ratio; LCL: lower confidence limit; UCL: upper confidence limit; WIC: Special Supplemental Nutrition Program for Women, Infants, and Children
